# Activity Screening of the Herb *Caesalpinia sappan* and an Analysis of Its Antitumor Effects

**DOI:** 10.1155/2021/9939345

**Published:** 2021-06-25

**Authors:** Yuan Li, Minghong Dong, Zijun Wu, Yongqi Huang, Haibing Qian, Cong Huang

**Affiliations:** ^1^Guizhou University of Traditional Chinese Medicine, Guian District, Guiyang 550025, China; ^2^Tianjin University of Traditional Chinese Medicine, Jinghai District, Tianjin 301699, China; ^3^Guizhou Province Key Laboratory of Prescription and Syndrome Pharmacology in Chinese Medicine, Guian District, Guiyang 550025, China

## Abstract

**Aim:**

Traditionally, *C. sappan* medicine was the heartwood, which needs to be cut down as a whole. In this research, the antitumor activity and mechanisms of the leaves and stems were compared with the roots of *Caesalpinia sappan*; it was in order to investigate whether stems and leaves of *C. sappan* could be used to replace heartwood for antitumor treatment, thereby reducing resource destruction.

**Methods:**

MTT assays were used to identify the active sites of *C. sappan* based on the application of human liver cancer (HuH-7) cells. High-performance liquid chromatography (HPLC) was used to analyze polar extracts. We also established a H22 hepatoma-bearing mouse model by administering intraperitoneal injections of petroleum ether extracts from the leaves and stems (SY②) at doses of 20 and 65 mg/kg. Mice in the i.g. group were administered intragastrically with the same extracts (at doses of 100 and 325 mg/kg) at the same time (12 days).

**Results:**

The antitumor site of *C. sappan* was the petroleum ether extract. The IC_50_ for the petroleum ether extract of roots (SG②) was 56.10 *μ*g/ml, while that for the leaves and stems (SY②) was 77.20 *μ*g/ml. Grey relational analysis indicated 11 active fraction peaks that were closely related to antitumor activity. The size of tumors in H22 hepatoma-bearing mice was reduced significantly in mice administered with petroleum ether extracts from the leaves and stems (inhibition rates of high doses were 55.31% and 60.56%). Fibrous tissue proliferation, inflammatory cell infiltration, tumor cell necrosis, and the expression of proliferating cell nuclear antigen (PCNA) and vascular endothelial growth factor (VEGF) were all lower than in the control group (VEGF *P* < 0.001 and PCNA *P* < 0.05).

**Conclusion:**

Petroleum ether extracts of the roots, leaves, and stems of *C. sappan* exhibit certain antitumor effects. Our data indicate that the mechanisms underlying these effects may relate to a reduction in the expression of PCNA and VEGF and the inhibition of angiogenesis. Our findings indicate that we can expand the medicinal use of *C. sappan* to the leaves and stems, thus improving resource utilization and reducing resource damage.

## 1. Introduction

As a common Hmong medicine, *Caesalpinia sappan* is the dried heartwood of a legume used in Guizhou folk herbal medicine for the treatment of tumors. The roots and bark of *C. sappan* were listed as sources for a herbal drug in the 2019 edition of *Quality Standards for Chinese Medicinal Materials and Ethnic Medicinal Materials in Guizhou Province*. The main ingredients of *C. sappan* are flavonoids, diterpenes, lactones, coumarins, and amides. Recent research on *C. sappan* found that a crude water extract of *C. sappan* can inhibit a variety of cancer cells or induce their apoptosis [1–3], thus demonstrating the value of further development and utilization. Other research showed that an ethyl acetate extract of *C. sappan* had a significant inhibitory effect on the growth of HGC-27 cells [4]. Protosappanin B has been shown to exert an inhibitory effect on the proliferation of BTT, T24, HeLa, and SW480 tumor cells [5]. In addition, brazilin has been shown to significantly inhibit the growth and proliferation of human bladder T24 cancer cells; the mechanism involved may be related to the upregulation of C-Fos expression [6]. Brazilin not only inhibits the proliferation of skin cancer cells (A431, BCC, SCC25, and A375) but also can also promote apoptosis in skin cancer cells by activating the expression of caspase-3 [7]. A previous study of the effect of brazilin on the inhibition of growth and the induction of apoptosis in human glioblastoma U87 cells showed that brazilin promotes cell apoptosis by increasing the cleavage of PARA and by downregulating the expression levels of caspase-3 and caspase-7 [8]. In another study, investigating the invasion of mouse liver cancer H22 cells, both protosappanin B and mitomycin were found to increase the survival time of Kunming mice [9]. Brazilin was also shown to exhibit a potential inhibitory effect on the tumorigenicity of mouse lung cancer A549-FIG cells in BALB/c nude mice by inhibiting the phosphorylation of barrier-to-autointegration factor (BAF) [10].

Traditionally, the roots and trunks of *C. sappan* are used to make medicinal extracts. However, this practice causes irreversible damage to the trees and severe damage to the resource itself. There are no reports in the existing literature that compare extracts made from the roots of *C. sappan* with those made from the leaves and stems with regard to anticancer effects. In the present study, we compared the efficacy of petroleum ether extract prepared from the roots with leaves and stems of *C. sappan* to inhibit the growth of human liver cancer (HuH-7) cells and as an antitumor therapy for H22 tumor-bearing mice. In this paper, we provide experimental evidence that supports the preparation of medicinal extracts from the stems and leaves of *C. sappan*, thus expanding the way this resource is used and providing options to prevent damage from being incurred by these valuable trees.

## 2. Materials and Methods

### 2.1. Drugs and Reagents

Antibodies(PCNA (clone VG1) and VEGF (clone VG1)) were purchased from DAKO Company. Trypsin (batch no. SH30042.01), DMEM (batch no. NXKO731), and FBS (batch no. NXCO582) were purchased from HyClone. Fluorouracil was acquired from Tianjin Jinyao while acetonitrile (no. 20121018), methanol (batch no. 20120201), dimethyl sulfoxide (DMSO, batch no. 2012062), and phosphoric acid were purchased from Tianjin Kemiou Company. Epirubicin was provided by the Beijing Botai Company. Paraformaldehyde tissue fixation solution was provided by the Second Affiliated Hospital of Guizhou University of Traditional Chinese Medicine.

### 2.2. Plant Material

*Caesalpinia sappan* (L.) Tod. plants were gathered from Ceheng County in Guizhou Province, China. The voucher specimen for *C. sappan* is GZTM 0018426 and is maintained in Guizhou University of Traditional Chinese Medicine, Guiyang.

First, we ground 1.0 kg of dry leaves and stems from *C. sappan* into a powder and then refluxed three times (75% ethanol, 5000 ml; 4 hours per reflux). The extracts were then mixed and concentrated; the ethanol extract yielded 143.0 g (SY①). The dregs were then dried and extracted with water three times. Next, we carried out six refluxes lasting 0.5 hours, 8 refluxes lasting 1 hour, and 12 refluxes lasting 2 hours. The solvent was then recovered so that we were left with the water extract; the yield of the water extract was 37.1 g (SY⑥). Different polar extracts were extracted from the ethanol extract, yielding 6.29 g of hydromethanolic extract (SY⑤), 3.15 g of n-butanol extract (SY④), 2.02 g of ethyl acetate extract (SY③), and 1.38 g of petroleum ether extract (SY②).

This procedure was then repeated to prepare extracts from the roots of *C. sappan* (1.0 kg). Using this strategy, we obtained 112.5 g of ethanol extract (SG①), 0.05 g of petroleum ether extract (SG②), 5.72 g of ethyl acetate extract (SG③), 1.12 g of n-butanol extract (SG④), 1.05 g of hydromethanolic extract (SG⑤), and 19.6 g of water extract (SG⑥).

### 2.3. Cell Lines

The H22 mouse liver cancer cell line and the human liver cancer HuH-7 cell line were purchased from the Shanghai Institute of Cell Research, Chinese Academy of Sciences. Dulbecco's Modified Eagle Medium (DMEM) was used to preserve cells (the medium contained penicillin G (100 U/ml), FBS, streptomycin (100 U/ml), and L-glutamine (2 mM) mg/ml).

### 2.4. Animals

Our experiments involved male and female Kunming mice (certificate no. SCXU (Qian) 2015-0004, weighing 18–22 g). The Local Committee on Animal Care and Use approved the experimental procedures. All experimentation was conducted according to guidelines provided by the Committee for Ethics in Animal Research (CEPA). Mice were housed under standard conditions in a breeding room (12 h dark/light cycle; 20–25°C; 60–75% humidity) with *ad libitum* access to food and water.

### 2.5. Determining the Effects of *C. sappan* Extracts on the Inhibition of Human Liver Cancer HuH-7 Cell Proliferation

The effects of *C. sappan* extracts were determined by MTT assays and by determining the IC_50_ of growth inhibition.

#### 2.5.1. MTT Assays

These assays were carried out as described previously [11, 12]. First, a cell suspension (1 × 10^5^ cells/ml) was added to a 96-well plate. Then, 100 *μ*l of each extract was placed in separate wells and placed in an incubator (5% CO_2_, 37°C) for 24 h. Water extracts and hydromethanolic extracts were dissolved with NS. DMSO (<0.1%) was used to aid the solubilization of n-butanol, ethyl acetate, and petroleum ether extracts. Administration groups were incubated with medicated serum (at doses of 100 *μ*g/ml, 20 *μ*g/ml, and 4 *μ*g/ml); three replicates were set up for each group. After 48 h of incubation, 50 *μ*l of MTT (5 mg/ml) was added to each well; this was followed by a 4 h incubation period. Next, the medium was removed and DMSO (150 *μ*l) was added to each well. Absorbance was then tested by a Multiskan FC Microplate Reader (Thermo Fisher Scientific) at 570 nm. These experiments were repeated twice. Data were then used to calculate the cell proliferation inhibition rate (IR_1_), as shown in the following equation:

(1) $$\begin{array}{c} {\text{IR}}_{1}=\left[\frac{\left(\text{OD value of control group}-\text{OD value of experimental group}\right)}{\text{OD value of control group}}\right]\times 100\%. \end{array}$$

#### 2.5.2. Determining the Antitumor Effects of Active Fractions

Based on preliminary experiments, the most effective active fractions were the petroleum ether extracts. Using equation (1), we determined the inhibition rate (IR_1_) of HuH-7 cells. Experimental groups featured a zero group (no cells), positive control groups (containing epirubicin at final concentrations of 20, 10, 5, 2.5, and 1.25 *μ*g/ml), two fraction administration groups (at doses of 200, 100, 50, 25, and 12.5 *μ*g/ml), and two control groups (PBS or DMSO).

### 2.6. High-Performance Liquid Chromatography (HPLC) Analysis

HPLC was used to investigate the stability, repeatability, and precision of all extracts. The RSD was less than 3.0%.

#### 2.6.1. Sample Solution Preparation

Samples of each polar extract (0.2 g) were weighed accurately and placed in different volumetric flasks (10 ml). A certain volume of methanol was then added into the volumetric flasks which were then shaken thoroughly. The samples were then sonicated for 30 mins and cooled at 20°C. The solutions were then filtered with a 0.45 *μ*m microporous membrane and used as test samples.

#### 2.6.2. Chromatographic Conditions

Extracts were analyzed by HPLC (Agilent 1100 with a quaternary pump and degasser, USA) in isocratic mode. All analyses were performed maintained at 25°C on an analytical Dima column (250 mm × 4.6 mm, 5 µm). The injection volume and flow rate were 10 *μ*l and 1.0 ml/min, respectively. Gradient elution was performed with mobile phases A (0.1% phosphoric acid), B (methanol), and C (acetonitrile) as follows: 0 min, 5% B and 5% C; 1 min, 15% B and 15% C; 60 min, 25% B and 25% C; 140 min, 33% B and 33% C; 270 min, 40% B and 40% C; 340 min, 45% B and 45% C; and 360 min, 50% B and 50% C. The detection wavelength was 240 nm.

### 2.7. The Mouse Tumor Model

H22 hepatoma-bearing mice were divided randomly into an intragastric (i.g.) low-dose group and high-dose group, a fluorouracil (FU) group, a control group, a low-dose group, and a high-dose group. On the basis of acute toxicity experiments, the SY② LD_50_ was 1961 mg/kg. The dose administered in the high-dose group (i.p.) was 1/30 of the LD_50_, the low-dose (i.p.) was 1/3 of the high dose. The dose administered to the high-dose group (i.g.) was 1/6 of the LD_50_ while the low-dose (i.g.) was 1/3 of the high-dose. At the end of the 12-day administration period, we measured pathological changes and measured the tumor weight. We also evaluated the expression of VEGF and PCNA in tumor tissues.

#### 2.7.1. Administration, Grouping, and Modeling

Ascites tumor-derived mice were produced by implanting H22 mouse hepatoma cells (0.2 ml/mouse) into the abdominal cavity. After 7 days, we used an injector to extract the nonbloody ascites which were then placed into an aseptic Petri dish [13]. Cells from the ascites were diluted to 1.0 × 10^7^ cells/ml. Then, 0.2 ml of ascites was inoculated into the left anterior axillary of each mouse (2.0 × 10^6^ tumor cells per mouse).

In total, 60 mice were grouped as follows (*n* = 10 per group): i.g. low-dose group (100 mg/kg), i.g. high-dose group (325 mg/kg), FU group (83 mg/kg), control group, i.p. low-dose group (20 mg/kg), and i.p. high-dose group (65 mg/kg). SY② was administered to each mouse from the third day after vaccination (at a dose of 0.2 ml/10 g). All mice were euthanized on day 13. The tumor inhibition rate (IR_2_) was then determined by weighing the tumors and applying the following equation:

(2) $$\begin{array}{c} {\text{IR}}_{2}=\left\{\frac{\left[\text{weight of tumors }\left(\text{control group}\right)-\text{weight of tumors }\left(\text{dosing group}\right)\right]}{\text{weight of tumors}\left(\text{control group}\right)}\right\}\times 100\%. \end{array}$$

#### 2.7.2. Pathological Analysis of Tumor Tissue

Tumor tissue was fixed, dehydrated, embedded, sectioned, and then stained with hematoxylin-eosin (HE). A light microscope was then used to determine whether any pathological changes had occurred. Lesions were then assessed using a scoring system (3 points: lesions occupy > 50%; 2 points: lesions occupy 30–50%; 1 point: lesions occupy < 30%) used to assess pathological changes. We also evaluated a range of indicators, including fibrous tissue proliferation, inflammatory cell infiltration, and tumor cell necrosis.

#### 2.7.3. Assays to Determine the Expression of VEGF and PCNA

Immunohistochemistry was carried out in accordance with the manufacturer's instructions using the following groupings: intragastric (i.g.), low-dose group, i.g. high-dose group, fluorouracil (FU) group, control group, intraperitoneal injection (i.p.) high-dose group, and i.p. low-dose group.

Mice were sacrificed after 13 days of administration. The tumor tissues were then soaked, dehydrated, embedded, deparaffinized, and sectioned at 4 *μ*m. The sections were then incubated with VEGF, washed with PBS, soaked in 0.3% H_2_O_2_ buffer (30 min), washed, incubated in PBS (10 min), and then incubated with primary antibody for 20 min at 4°C. Sections were then washed with PBS (10 min), incubated with EnVision™ for 20 min, and then incubated with a color source substrate solution incubated for 20 min. Immunopositivity was indicated as brown/yellow particles located in the cytoplasm. Five dense areas of tissues (100 fields) of neovascularization were selected [14] and the number of microvessels in each view field-of-view (200 fields) were counted.

Other portions of the tissue were immunohistochemically stained for PCNA. PCNA-positive protein (brown/yellow particles) were located in the nucleus. Five tissue areas were selected at random to count PCNA-positive tumor cells (400 fields).

### 2.8. Statistical Methods

Data were analyzed with SPSS version 23.0. *P* values of 0.05 or less were considered to be statistically significant. Data are presented as mean ± standard deviation (SD) for individual groups (*n* ≥ 3).

## 3. Results

### 3.1. *C. sappan* Extracts Inhibited the Proliferation of HuH-7 Cells

**Figure 1:**
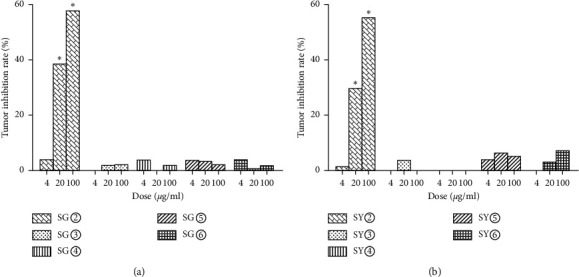
The inhibitory effect of *C. sappan* extracts. (a) The roots of *C. sappan.* (b) The leaves and stems of *C. sappan.* Compared with the DMSO control group, ^*∗*^*P* < 0.05.

**Figure 2:**
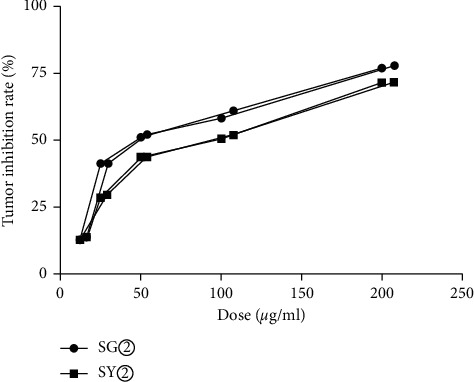
Inhibition curves for SY② and SG②.

**Figure 3:**
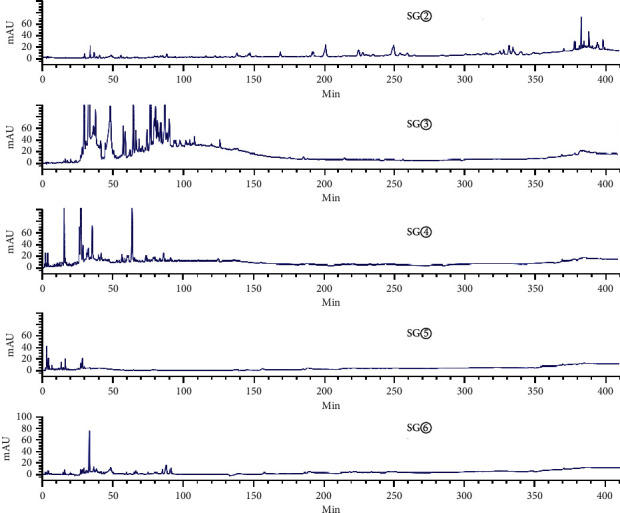
HPLC chromatograms for water extract (SG⑥), hydromethanolic extract (SG⑤), n-butanol extract (SG④), ethyl acetate extract (SG③), and petroleum ether extract (SG②), prepared from the roots of *C. sappan*.

The antitumor results for *C. sappan* extracts are summarized in Table 1. Analysis showed that SY② and SG② significantly inhibited the proliferation of HuH-7 cells (Figures 1–3). These data indicated that *C. sappan* petroleum ether extracts (SY② and SG②) might be the most effective active fractions against tumors. The mean of the IC_50_ values for the duplicated experiments was taken as the final index for inhibition. The IC_50_ value of epirubicin, SY②, and SG②, were 5.11 *μ*g/ml, 77.20 *μ*g/ml, and 56.10 *μ*g/ml (Table 2). The IC_50_ of SG② was smaller than that of SY②. Furthermore, the inhibitory effect of SY② was stronger than SG②.

### 3.2. HPLC Analysis

**Figure 4:**
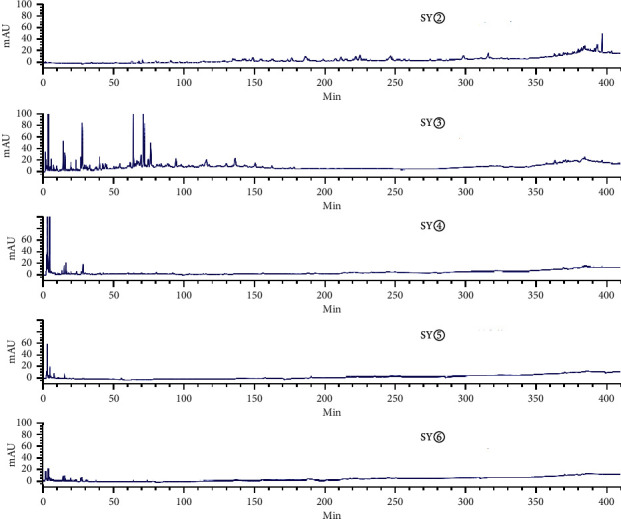
HPLC chromatogram for water extract (SY⑥), hydromethanolic extract (SY⑤), n-butanol extract (SY④), ethyl acetate extract (SY③), and petroleum ether extract (SY②) prepared from the stems and leaves of *C. sappan*.

Figure 3 shows an HPLC chromatogram of the polar extracts prepared from *C. sappan* roots. Compared with SG②, between 100 min and 410 min, SG④, SG⑤, and SG⑥ showed no chromatographic peaks. From 0 min to 130 min, SG③ had the most chromatographic peaks. This data implies that the roots had more components than the stems and leaves. The chromatogram of SY② was significantly different from the other four samples (Figure 4). When compared with SY③, SY④, SY⑤, and SY⑥, SY② showed no common peaks.

**Figure 5:**
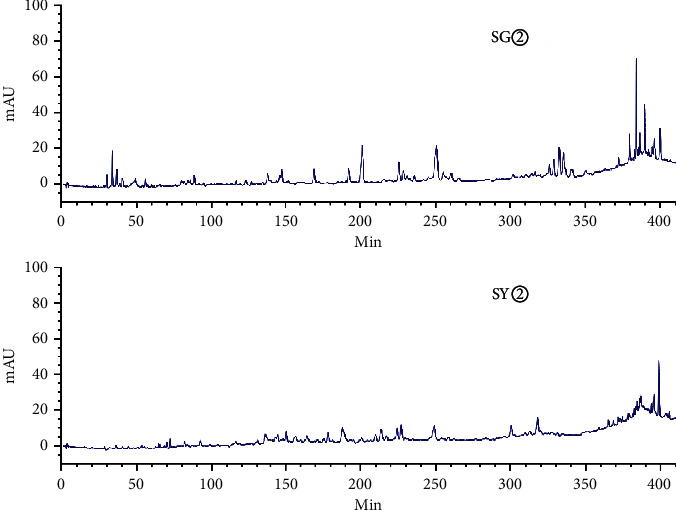
A comparison of the petroleum ether extracts (SG② and SY②).

**Figure 6:**
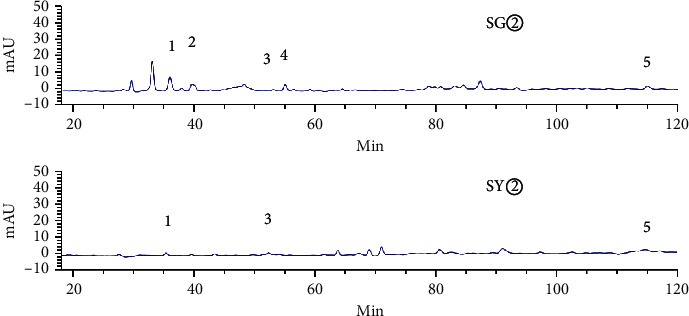
Common peaks identified by HPLC for SG② and SY② (1–5).

**Figure 7:**
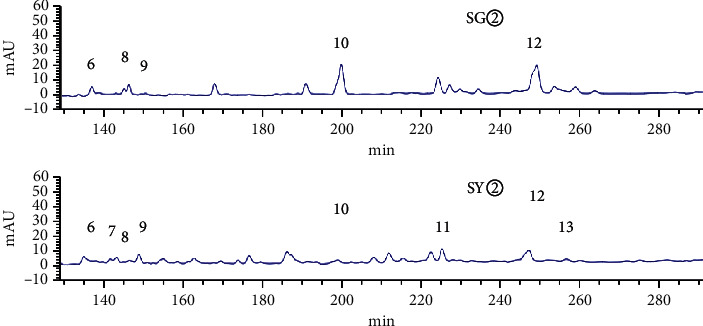
Common peaks identified by HPLC for SG② and SY② (6–13).

**Figure 8:**
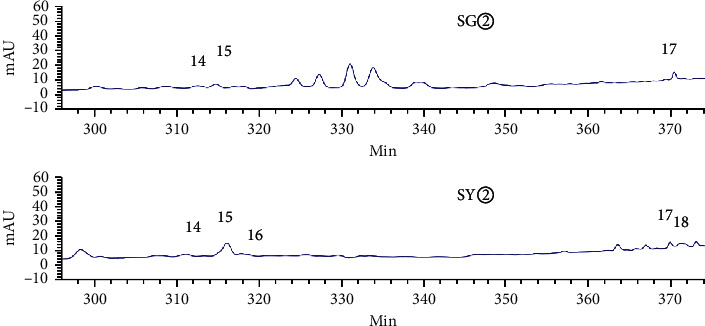
Common peaks identified by HPLC for SG② and SY② (14–16).

**Figure 9:**
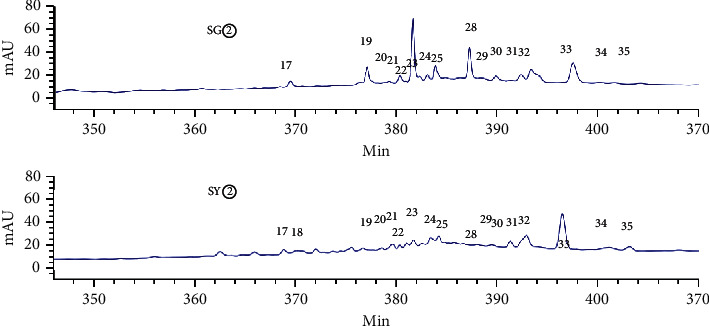
Common peaks identified by HPLC for SG② and SY② (17–35).

HPLC analysis found that 15906.1 and 13736.9 were the total peak areas for the active fractions (SG② and SY②). There were 26 common peaks (Figures 5–9).

### 3.3. Grey Relational Analysis

Analysis showed that the most effective active fractions were the petroleum ether extracts of *C. sappan* (SY② and SG②). The IC_50_ for HuH-7 cells was used as the pharmacodynamic index for active chemicals. We then used Grey correlation to analyze the association between antitumor activity and common peaks [15–17]. Analysis showed that the common peaks were correlated to antitumor activity (correlation coefficients > 0.6): 31 > 3 > 33 > 5 > 29 > 35 > 6 > 30 > 15 > 28 > 26 peaks (Table 3).

### 3.4. Analysis of Tumor Weight

**Figure 10:**
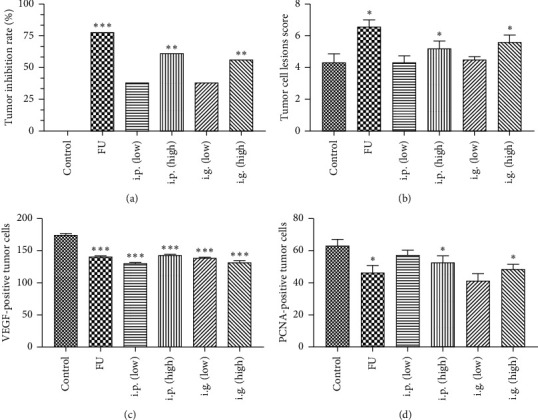
SY② antitumor effects in H22 hepatoma-bearing mice. All mice were administered for with SY② 12 days by the intragastric route (100 and 325 mg/kg), by intraperitoneal injection (20 and 65 mg/kg), and FU (83 mg/kg). (a) Tumor inhibition rate obtained by evaluating the weight of tumors, ^*∗∗*^*P* < 0.01 and ^*∗∗∗*^*P* < 0.001 versus the control group. (b) Pathological scores for tumors (HE staining), ^*∗*^*P* < 0.05 versus the control group. (c) VEGF expression, ^*∗∗∗*^*P* < 0.001 versus the control group. (d) PCNA expression, ^*∗*^*P* < 0.05 versus the control group.

Table 4 and Figure 10(a) show that tumor weight in the FU group was significantly reduced compared with the control group (*P* < 0.001); the high-dose i.p. group (*P* < 0.001) and i.g. group (*P* < 0.05) showed similar effects. This suggested that a high dose (i.g. and i.p.) could inhibit tumor growth. However, the weight of tumors in the low-dose groups (i.g. and i.p.) were not significantly different from the control group.

### 3.5. Pathological Scores of Tumors (HE Staining)

**Figure 11:**
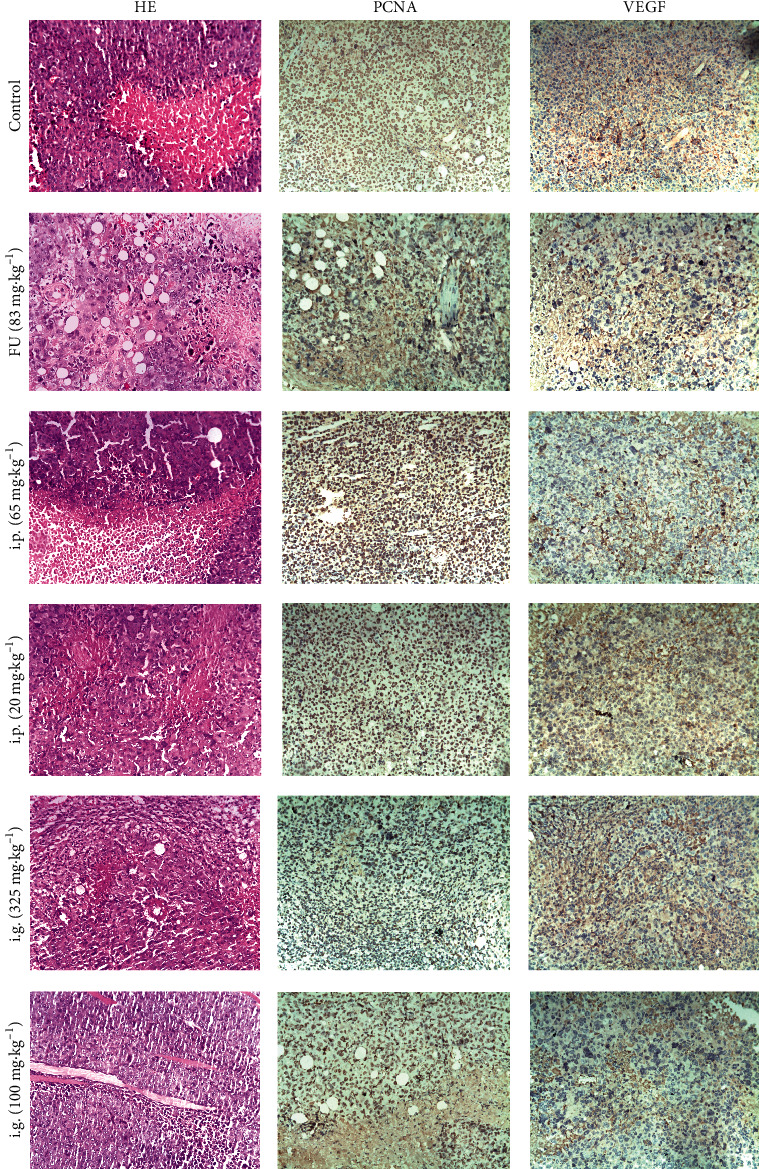
The antitumor effects of SY② in H22 hepatoma-bearing mice. All mice were administered with SY② for 12 days by the intragastric route (100 and 325 mg/kg), intraperitoneal injection (20 and 65 mg/kg), and by FU (83 mg/kg). Light microscopy 200x, bars = 100 *μ*m, for VEGF, PCNA, and HE staining.

In the control group, tumor cells showed deep chromatin staining, irregular shapes, and polymorphism (Figures 10(b) and 11). In the treatment groups, tumor cells showed differing extents of necrosis, fibrous tissue proliferation, and the infiltration of inflammatory cells. In the low-dose groups, there was no significant effect on pathological changes related to tumors. In the high-dose groups, there was a significant reduction in the lesion score (*P* < 0.05) (Table 5 (a)).

### 3.6. VEGF Expression

Light microscopy showed that VEGF-positive products were localized in the cytoplasm and expressed in the cytoplasm of the arterial endothelial cells, small veins, and capillaries in tumors (Figures 10(c) and 11). Angiogenesis in tumor tissue was significantly inhibited in the low-dose, high-doses, and FU injection groups (*P* < 0.001) (Table 5 (b)).

### 3.7. PCNA Expression

Light microscopy detected PCNA-positive products in the nuclei (Figures 10(d) and 11). The expression of PCNA in the high-dose groups was significantly lower (*P* < 0.05) than in the control group (Table 5 (c)). Tumor cell proliferation was significantly inhibited in the i.p. and i.g. high-dose and FU injection groups.

## 4. Discussion

In this study, HPLC chromatograms demonstrated that there were similar chemical components in the roots, stems, and leaves. We also used the Grey relational analysis to evaluate the relative contribution of each component to tumor suppression; 11 common peaks were shown to be correlated with tumor inhibition activity. These components exhibited a congenerous antitumor effect that was consistent with the advantages of traditional Chinese medicine (multicomponents and multitargets) [18]. Analysis showed that a *C. sappan* represented a key resource for a large number of antitumor compounds, thus providing a useful foundation for future research focusing on identifying compounds with antitumor activity.

Tables 1 and 2 show that the petroleum ether extract of *C. sappan* exhibited the best antitumor activity. The petroleum ether extract of leaves and stems (SY②) significantly inhibited the proliferation of tumor cells with an IC_50_ of 77.20 *μ*g/ml; this was even better than the roots, the resource that is traditionally used to prepare extracts (roots: SG②; IC_50_ = 56.10 *μ*g/ml). Therefore, it follows that leaves and stems could also be used as a medicine for antitumor therapy. The Grey relational analysis indicated that 11 active fractions exhibited common peaks (31#, 3#, 33#, 5#, 29#, 35#, 6#, 30#, 15#,28#, and 26#) that were closely related to antitumor activity. This foundation was used in the next step of our research which focused on extracting, isolating, and identifying compounds with antitumor activity.

There was a significant reduction in the tumor tissue of H22 hepatoma-bearing mice in response to high-dose administration (Table 4). Tumor cells in the control group showed deep chromatin staining, irregular shapes, and polymorphism (Figures 10(b) and 11). In the treatment groups, the tumor cells exhibited differing degrees of necrosis, fibrous tissue proliferation, and the infiltration of inflammatory cells. The lesion score for tumor cells in the high-dose groups was significantly lower than in the other groups, thus showing that the petroleum ether extract of leaves and stems had a reliable and precise antitumor effect *in vivo*. Compared to other groups, the high-dose treatment groups showed reduced fibrous tissue proliferation, inflammatory cell infiltration, and necrosis in tumor cells; these groups also showed reduced expression of PCNA and VEGF.

Excessive proliferation and distant metastasis are important biological characteristics of malignant tumors. Therefore, developing methods to inhibit the proliferation of tumor cells and prevent their distant metastasis has become the main focus of clinical treatment. PCNA, VEGF, and other factors play important roles in promoting tumor proliferation and metastasis, PCNA is a cyclin, an accessory protein for cell DNA polymerase that exists in each developmental stage of tumor cells and the normal proliferation cycle of cells. However, PCNA is expressed at low levels in the G0 and G1 phases. The expression of PCNA begins in the G1 phase and reaches a peak in the S phase but decreases significantly in the M and G2 phases. This means that the changes in PCNA expression are consistent with DNA synthesis and are one of the most effective markers for detecting cell proliferation. PCNA expression can directly reflect the proliferation of tumor cells. The higher the expression of PCNA, the more active the state of cell proliferation; therefore, PCNA is regarded as being a useful indicator of the proliferation status of a cell [19–21]. Many studies have shown that the metastasis and growth of tumor tissue are dependent on a sufficient nutrient supply, new blood vessels, and oxygen. On the other hand, due to the high permeability of new blood vessels, tumor cells can readily pass through and spread. Revascularization provides conditions for infiltration and metastasis [22]. The VEGF family are members of the receptor tyrosine kinase (RTK) family and play a key role in tumor angiogenesis. Consequently, VEGF is one of the most important inducers of angiogenesis and increases vascular permeability. Once VEGF binds to its corresponding receptor, it stimulates the proliferation of vascular endothelial cells in the tumor and increases vascular permeability to provide a suitable basis for tumor infiltration and metastasis; consequently, VEGF is one of the main targets for tumor therapy [23].

We showed that the petroleum ether extract of leaves and stems could significantly inhibit the proliferation of tumor cells *in vivo*. Our data also demonstrated that, within the treatment groups, each dose inhibited the proliferation of tumors and that there was a significant reduction of tumor size compared to the model group; there was also a reduction in the extent of pathological changes. Immunohistochemical staining showed that the number of PCNA-positive cells within the tumor tissue was lower than in the model group, thus indicating that the mechanism underlying the inhibitory effect of SBLPE on tumor proliferation may be related to the inhibition of high PCNA expression. SBLPE was shown to inhibit the expression of VEGF in tumor tissues in an effective manner and that VEGF was the strongest angiogenesis-promoting factor and played a significant role in promoting the proliferation and metastasis of tumor tissues. Our data showed that SBLPE inhibited the expression of VEGF in vascular endothelial cells. Furthermore, our data indicated that SBLPE inhibited the growth and angiogenesis of H22 tumors in mice and helped to inhibit the distant metastasis of tumor cells through immature blood vessels. Combined with the expression of VEGF in SBLPE-treated tumor tissues, our data indicated that SBLPE may inhibit the growth and angiogenesis of H22 tumors by inhibiting the secretion of VEGF by tumor cells, therefore inhibiting the metastasis and proliferation of tumor cells.

The mechanisms underlying tumor cell proliferation and metastasis are complicated. Our research only investigated the mechanisms underlying anti-H22 tumor effects by evaluating antitumor growth and the growth of microvessels. Further studies are now needed to confirm these mechanisms and identify associated pathways. However, it is important to note that SBLPE is still a mixture. Further studies are now needed to identify the effective ingredients in SBLPE. However, our results provide a meaningful basis for the further identification of effective substances with antitumor effects.

## 5. Conclusions

In this paper, we demonstrated, for the first time, that petroleum ether extracts prepared from the leaves and stems of *C. sappan* can inhibit the proliferation of tumor cells. Compared with the resources that are traditionally used to prepare medical extracts (the roots and the trunk), the use of the stems and leaves would cause less damage to valuable resources. Therefore, our data indicates that the range of resources used to prepare extracts could be expanded to the leaves and stems, thus protecting valuable *C. sappan* resources.

Petroleum ether extracts prepared from the leaves and stems of *C. sappan* reduced the expression of PCNA and VEGF in the tumors of H22 hepatoma-bearing mice. Grey relational analysis indicated that the 11 common peaks of active fractions were closely related to antitumor activity. Our data provide a useful foundation for the extraction, isolation, and identification of compounds with antitumor activity.

## Figures and Tables

**Table 1:** The inhibitory effect of *C. sappan* extracts (*n* = 6, mean ± SD).

Group	Dose (*μ*g/ml)	OD value	IR_1_ (%)	Group	Dose (*μ*g/ml)	OD value	IR_1_ (%)
NS	—	0.2353 ± 0.1087	0	NS	—	0.2307 ± 0.1079	0

DMSO	—	0.2018 ± 0.0572	0	DMSO	—	0.1963 ± 0.0530	0

SY②	100	0.0900 ± 0.0466^*∗*^	55.4	SG②	100	0.0830 ± 0.0656^*∗*^	57.7
	20	0.1412 ± 0.0468	30.0		20	0.1206 ± 0.0284^*∗*^	38.6
	4	0.1983 ± 0.0613	1.7		4	0.1880 ± 0.0366	4.2

SY③	100	0.2028 ± 0.0610	0	SG③	100	0.1912 ± 0.0548	2.6
	20	0.1938 ± 0.0536	4.0		20	0.1918 ± 0.0579	2.3
	4	0.2037 ± 0.0803	0		4	0.2148 ± 0.0531	0

SY④	100	0.2035 ± 0.0600	0	SG④	100	0.1920 ± 0.0515	2.2
	20	0.2083 ± 0.0661	0		20	0.2142 ± 0.0578	0
	4	0.2090 ± 0.0817	0		4	0.1883 ± 0.0564	4.1

SY⑤	100	0.2222 ± 0.0975	5.6	SG⑤	100	0.2248 ± 0.0959	2.6
	20	0.2193 ± 0.0844	6.8		20	0.2225 ± 0.0892	3.6
	4	0.2253 ± 0.0835	4.2		4	0.2215 ± 0.0866	4.0

SY⑥	100	0.2177 ± 0.0971	7.5	SG⑥	100	0.2257 ± 0.0917	2.2
	20	0.2270 ± 0.0989	3.5		20	0.2287 ± 0.0990	0.9
	4	0.2387 ± 0.1039	0		4	0.2210 ± 0.0825	4.2

Note: compared with the DMSO control group, ^*∗*^*P* < 0.05.

**Table 2:** The inhibitory effect of SY② and SG② (*n* = 3, mean ± SD).

Group	Dose (*μ*g/ml)	First experiment			Second experiment		
		OD value	IR_1_ (%)	IC_50_ (*μ*g/ml)	OD value	IR_1_ (%)	IC_50_ (*μ*g/ml)
PBS	—	0.1838 ± 0.0082	0	—	0.1860 ± 0.0068	0	—

DMSO	—	0.1752 ± 0.0028	0	—	0.1790 ± 0.0104	0	—

SY②	200	0.0500 ± 0.0164	71.5	76.6	0.0523 ± 0,0169	71.6	77.8
	100	0.0843 ± 0.0460	51.9		0.0912 ± 0.0470	50.5	
	50	0.0990 ± 0.0380	43.5		0.1007 ± 0.0437	43.6	
	25	0.1247 ± 0.0190	28.9		0.1300 ± 0.0251	29.5	
	12.5	0.1510 ± 0.0318	13.9		0.1607 ± 0.0267	12.8	

SG②	200	0.0390 ± 0.0204	77.7	56.05	0.0413 ± 0.0212	76.9	56.14
	100	0.0733 ± 0.0458	58.2		0.0700 ± 0.0396	60.9	
	50	0.0841 ± 0.0338	52.0		0.0877 ± 0.0360	51.0	
	25	0.1027 ± 0.0317	41.4		0.1053 ± 0.0291	41.2	
	12.5	0.1520 ± 0.0211	13.2		0.1560 ± 0.0250	12.8	

Epirubicin	20	0.0289 ± 0.0112	83.5	5.42	0.0273 ± 0.0103	84.7	4.79
	10	0.0667 ± 0.0103	61.9		0.0657 ± 0.0104	63.3	
	5	0.0898 ± 0.0189	48.7		0.0839 ± 0.0183	53.1	
	2.5	0.1135 ± 0.0121	35.2		0.1102 ± 0.0118	38.5	
	1.25	0.1563 ± 0.0134	10.7		0.1545 ± 0.0133	13.6	

**Table 3:** Correlation between common antitumor activity and peaks of activity.

Peak	Correlation
1	0.355
3	0.889
5	0.736
6	0.670
8	0.407
9	0.588
10	0.354
14	0.565
15	0.629
17	0.465
19	0.499
20	0.564
22	0.484
23	0.477
24	0.441
25	0.507
26	0.622
27	0.399
28	0.626
29	0.699
30	0.667
31	1.000
32	0.593
33	0.831
34	0.489
35	0.685

**Table 4:** Tumor weight and IR_2_ (*n* = 10, mean ± SD).

Group	Administration routes	Dose (mg/kg)	Tumor weight (g)	IR_2_ (%)
Control group	i.p. + i.g.	—	1.607 ± 0.6241	—

FU group	i.p.	83	0.352 ± 0.1862^*∗∗∗*^	78.09

i.p. group	i.p.	65	0.624 ± 0.4143^*∗∗*^	61.16
		20	1.000 ± 0.6528	37.77

i.g. group	i.g.	325	0.707 ± 0.6373^*∗∗*^	56.01
		100	0.995 ± 0.7709	38.08

Note: compared with the control group, ^*∗*^*P* < 0.05, ^*∗∗*^*P* < 0.01, and ^*∗∗∗*^*P* < 0.001.

**Table 5:** Pathological changes in tumor tissue and the expression of VEGF and PCNA factors (*n* = 10, mean ± SD).

Group	Dose (mg/kg)	Scores^a^	VEGF^b^	PCNA^c^
Control group	—	4.31 ± 0.54	174.915 ± 1.7091	63.13 ± 3.78

FU group	83	6.57 ± 0.43^*∗*^	141.21 ± 0.9003^*∗∗∗*^	46.12 ± 4.45^*∗*^

i.p. group	65	5.20 ± 0.46^*∗*^	144.189 ± 1.0205^*∗∗∗*^	52.80 ± 3.90^*∗*^
	20	4.30 ± 0.42	131.066 ± 0.9191^*∗∗∗*^	57.40 ± 2.82

i.g. group	325	4.50 ± 0.17^*∗*^	132.924 ± 1.7579^*∗∗∗*^	48.70 ± 2.62^*∗*^
	100	5.60 ± 0.43	139.358 ± 1.3292^*∗∗∗*^	41.10 ± 4.62

^a^Tumor cell lesion score (HE staining). ^b^VEGF-positive tumor cells. ^c^PCNA-positive tumor cells. Compared with the control group, ^*∗*^*P* < 0.05 and ^*∗∗∗*^*P* < 0.001.

## Data Availability

All data for this project are available from the corresponding author.
